# Influence of Strain Route Changes on the Microstructure and Mechanical Properties of CuZn36 Alloy during Cross Channel Extrusion CCE

**DOI:** 10.3390/ma15031124

**Published:** 2022-01-31

**Authors:** Radosław Łyszkowski

**Affiliations:** Faculty of Advanced Technology and Chemistry, Military University of Technology, 00-908 Warsaw, Poland; radoslaw.lyszkowski@wat.edu.pl

**Keywords:** strain route, cross-channel extrusion (CCE), severe plastic deformation (SPD), structure and properties

## Abstract

This study evaluates the impact of changing the deformation routes of the extrusion process in a cross-shaped die (CCE) on the structure and properties of a CuZn36 alloy (% at.). Samples with dimensions of Ø8 × 36 mm were subjected to extrusion at room temperature according to two variants: straight extrusion in the A route (2-, 4-, 8- and 12-pass) and extrusion with interoperative rotation by 90° in the B_C_ route (2- and 4-pass). The improvement of strength properties was obtained as a result of grain fragmentation in the CCE process. Changes in the microstructure were observed using a light microscope, and mechanical properties were measured in the microhardness test and a static tensile test. The obtained results showed that the mechanical properties of the alloy depend on the number of passes and the material deformation route. This observation was related to the fragmentation of its structure and strengthening, which resulted in changes in its properties. The highest strength was characterized by the material pressed four times with the rotation of 90° (B_C_ route), whose properties were comparable and even slightly better than the material squeezed twelve times without rotation (A route).

## 1. Introduction

The development of technology and the increasing requirements for structures in the construction and machine industry have resulted in the growing interest and demand for newer materials with better strength properties. One of the ways to improve the mechanical properties of materials is to reduce the grain size by plastic working. Along with reducing grain, the materials show much better mechanical properties [1]. The fragmentation of the material structure causes an increase in the number of grain boundaries, which become a barrier to dislocation movement. This strengthens the material and leads to an increase in mechanical properties such as hardness and tensile strength. In ultrafine-grained materials (UFGs), their increase is even more than twice that of their coarse-grained counterparts. Therefore, special attention should be given to methods using severe plastic deformation (SPD) [2,3].

By limiting the possibility of free movement deformation and complex stress states, these methods allow us to obtain results not achievable by classical plastic working, making them quite attractive in terms of application [4,5]. The effectiveness of grain refinement depends on several factors, including the basic deformation mechanism influencing the value of unit deformations and the possibility of multiple repetitions of the basic deformation pattern determining the total deformation value and changing the deformation route by appropriate rotation of the workpiece samples.

Among SPD techniques, the high-pressure torsion process is considered the most efficient [6]. In this method, the deformation is caused by shear stresses supported by compressive stresses. This allows obtaining very high plastic strains without breaking the materials and nanomaterials [7]. However, the process depends on the dimensions of the disk sample, which are unfortunately relatively small. Accumulative roll bonding (ARB) is a relatively new SPD process where, as a result of limited rolling combined with stacking the charge, it is possible to obtain significant deformation of large and bulk samples [8,9]. This process leads to the formation of the UFG structure in the material. Due to rolling, the product range is limited essentially to plates, sheets, and strips [10].

The most common SPD technique is based on the side extrusion process [11]. The method consists of squeezing the material through the channel with the angular change of its route. The deformation occurs as a result of simple material shearing in the refraction plane. Unlike the previously described techniques, deformation in the limited volume of the die channel does not pose the problems mentioned above and is predictable. Relatively simple tooling is therefore considered an advantage. Two groups of processes were selected in this study, extrusion in a single channel and in systems of intersecting channels. The first includes the equal channel extrusion (ECAE) process, also known as equal channel angular pressing (ECAP) [4].

It is a relatively simple technology that allows us to obtain materials with UFG structures [12]. The products are generally in the shape of bars or flat bars. During processing by ECAP/E, the metal billet is pressed through a die with a sharp angle bent (30–120°) constant-cross-sectional channel. As a result, the billet does not change its dimensions during deformation, which allows this process to be repeated many times and obtain very large total deformations. Despite the undoubted advantages, the method also has drawbacks related to the friction of the material against the channel walls and the so-called “corner gap” where the deformation of the charge is strongly disturbed [13]. These problems can be reduced by using movable die walls, but this complicates the construction of the device [14,15].

**Figure 1:**
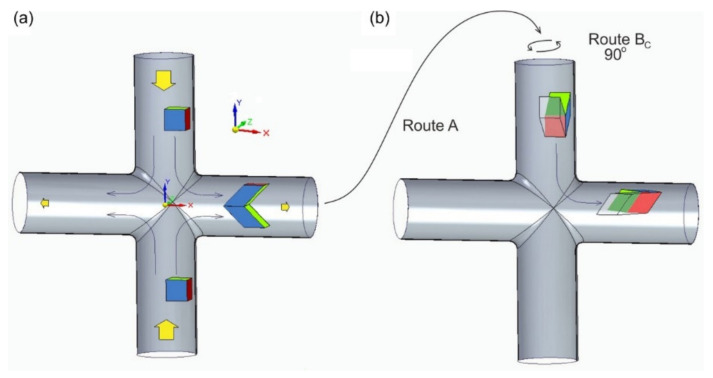
Schematic illustration of the cross channel extrusion CCE process (**a**) and basic processing routes (**b**) with distortion patterns.

The second group consists of techniques based on developing a single angular channel to multichannel systems. An example of this is the T-shaped ECAP method [16,17], where the channel takes shape “┬” or forms an intersecting pattern “┼” as in cross channel extrusion CCE (Figure 1) [18]. The mentioned aspect of the production of UFGs in the structure relates to the possibility of obtaining high values of total deformation by repeating the basic process. In a method such as HPT, this may be a continuous process essentially related to the number of revolutions of the sample. However, in most techniques, this requires the removal of a processed billet of material and reloading it into the device. Only a few of them provide the possibility of repeating the deformation by reversing the flow direction. Not having to remove the sample from the instrument significantly increases the productivity of the process [19].

This study evaluates the third aspect of material processing with SPD techniques. When a billet is reloaded into the fixture, it is possible to position it to undergo deformation with the same (basic) or rotated stress pattern. This process is called “changing of route deformation” [2]. It is conducted by rotating the sample in relation to the main or additional axes of the system between successive cycles of its processing and then set about a deformation according to routes A, B, C, etc. The detailed characteristics of individual processing methods can be found in Valiev [4] and Segal [20]. Analyzing the macroscopic patterns of distortion, one can consider the influence of the processing route on the development of the material’s microstructure. Dynamically developing SPD techniques, as more than 120 types are listed in [21], entails specifying new distortion patterns and routes of deformation.

In the scheme according to route A, the sample is processed without rotation between successive passes, and the shearing plane and direction are the same in both even and odd-numbered passes. Route B consists of rotating the sample by an angle of 90° around the extrusion axis, wherein in the B_A_ variant, the specimen is rotated in an alternating clockwise direction and the B_C_ variant, only unidirectionally. For both variants, shearing occurs at different shearing planes for each pass, repeated after two successive passes without any inversion in the shearing direction for the B_A_ route and after four passes with inversion of the shearing direction for B_C_. In route C, the specimen is rotated by 180° between subsequent passes, and the shearing plane is always the same in successive passes, but the shearing direction is inverted after each pass [11].

The material properties strongly depend on the direction of its processing [22]. For the samples oriented toward ED in relation to the extrusion direction of the plate from which they were taken, a higher hardening rate and less plastic flow were observed, while ND samples exhibited larger strains to fracture. In [23], it was shown that the introduction of a bend in the route in the accumulative angular drawing (AAD) method not only results in the possibility of deformation of the Ti alloy of the wire to a previously unattainable degree of ~0.5 but also an increase in its plasticity. The authors prove that the responsible change in the deformation path activates additional deformation modes.

The results of the asymmetric rolling (ASR) process show that it leads to deformation much more efficiently, causing severe deformation or microstructural evolution and improving the ductility of the alloy in comparison with samples deformed by the classical method of symmetric rolling (CR) [24]. By appropriately differentiating the ASR deformation paths, one can influence the deformation value and the material structure. This is due to the intersecting shear patterns of successive transitions favoring the formation of fine subgrains within the shear band [9,25]. Additionally, in the processing of flat elements, such as constrained groove pressing CGP [26] or repetitive corrugation and straightening by rolling (RCSR) [27], where, in subsequent operations the crossing strain paths create a specific reinforcement network, the UFG structure is formed, and the mechanical properties increase. As the number of passes increased, the strain increased and became more uniform despite the overall heterogeneity.

The literature expresses the view that processing with the ECAP method reduces the occurrence of casting defects, such as shrinkage cavities and the separation of second-phase particles by breaking and dispersing them in the alloy matrix, and affects grain refinement by breaking up coarse dendrites [28,29,30]. However, the route with which the sample re-enters the die in each pass had an impact on the obtained microstructure and its properties [15] due to the successive change of the shear plane.

Shaeri et al. [31] reported that ECAPed Al alloys in the A route showed an inhomogeneous microstructure with elongated grains in comparison with the specimen processed via the B_C_ route. Others [32] investigating the effect of the processing method on the grain size of Ti-Al metal composites noticed that the A route is more effective in refining the matrix than the C route. The authors of [33] came to similar conclusions. However, due to the three-dimensional deformation, this processing by the B_C_ route provides a finer microstructure compared to other routes [34].

Research on the impact of the ECAP deformation routes on the mechanical properties of Al-Si alloys showed that processing according to the B_C_ route leads to the location of deformation in the eutectic phases and a decrease in elongation to alloy fracture compared to the case of A [35]. In the study [36], it was reported that the deformation in the B route is more extreme than in the case of A and C and may lead to significant structural differences. However, the material was characterized by similar durability, regardless of the route variant adopted. Avvari et al. [37] reported that for an AZ31 alloy, B_C_-ECAP deformation was the most efficient in terms of deformation and microhardness increase, and the grain size after two passes was reduced by more than two times, regardless of the route type. Gao et al. [38] showed that after 4xECAP processing in B_C,_ it has a positive effect on the homogeneity of deformation of AA6063 alloy, noting that variant C is only slightly inferior in terms of strength and plasticity. Increasing the number of passes to 6–8 leads to a further decrease in the grain size and improvement of the homogeneity of the structure and only a slight increase in hardness and strength, with little preference for the B_C_ or C route in this regard [39,40,41]. Horita et al. [42] found that the UFG microstructure of pure aluminum after ten passes in the A route by ECAP was the same as after four passes in B_C_ route.

Interesting effects were noted when the extrusion method was combined with the cyclic change of the deformation path by the oscillating die, the so-called KOBO [43]. In this method, the oscillations force continuous changes in the deformation path and lead to an increase in the mechanical properties of the processed material [44]. Even more interesting results can be obtained when fundamentally different SPD processes are combined, e.g., ECAP with plane compression [45], rolling with extrusion (DRECE process) [46], or with shear, ECAP, and extrusion (CONFORM process) [47], but that is beyond the scope of this article.

It should also be added that repeating the deformation pattern may slightly weaken the material hardening effect in even passages. This is due to the opposite direction of deformation causing the Bauschinger effect [48]. Wang et al. [49] showed a decrease in the yield strength of AZ31 alloy samples when compression was replaced with stretching. Orlov et al. [50] found that the cyclical change in the direction of rotation in the HPT reduces the rate of formation of high-angle grain boundaries (HAGBs) and slows down the nanostructure due to the decrease in dislocation density [51].

A relatively recently appeared method known for SPD techniques is cross channel extrusion CCE [18] or cross-equal channel angular pressing C-ECAP [52]. The main advantages of this method are the possibility of obtaining a strong deformation of solid material in single production cycles and its multiple repetitions without removing the sample from the instrument by reversing the extrusion direction. This makes it a prospective method for the industrial production of a suitable volume of fine material.

FEM numerical simulations [53] and experimental research [54] revealed that in the CCE method, the material is deformed due to the combination of two processes. The first is the shear of the material as it passes through the angle and is analogous to the basic ECAP system. The second is related to hydrostatic stresses caused by the two-sided pressing of the billet in the input channel (along the Y-axis). They also occur in the ECAP method, but their influence on the deformation is negligible. This is confirmed by the already mentioned problem with the “corner gap”, where, in the outer corner of the die, material tears off the walls of the channel [55]. The opposing movement of the material in the CCE causes that when it passes through the kink of the channel, it does not rub against its bottom, as in the case of ECAP, because the pressure forces cancel each other out. In the center of the die, at the intersection of both channels, a very strong reorganization of the material takes place under the influence of the dominant triaxial compression. Two particles that are still adjacent to each other in the input channel can be plastically separated (without losing the cohesion of the material) and moved to the output channel in opposite directions. This is illustrated by the arrows in Figure 1a. As a result, what is not observed in the ECAP is the formation of a zone of very strong deformations located along the longitudinal Axis X. While the first of the mentioned mechanisms causes a unit deformation ε ≈ 1–1.1, this combination of both results in a deformation exceeding even ε ≈ 5. There is a significant differentiation of the material structure and its properties, divided into two or even three distinct zones. Subsequent sample passages through the matrix channel lead to an increase in the deformation and its propagation in the billet volume.

However, over decades since Chou patented the CCE method [18], the influence of the way the sample moves through the cross pattern of the matrix channels with its rotation, i.e., the change in the deformation route, has not yet been studied. In addition, even today, more than 30 years after Segal and Valiev’s first work, little information is available regarding the effect of processing routes in complex SPD systems on the microstructure and material properties. Therefore, the main goal of this study was to implement systematic research on the impact of changing the deformation route, particularly in the CCE method, on the microstructure and mechanical properties of the tested material. The results of this work, apart from general cognitive knowledge, will be helpful in the implementation of this method for the production of UFG alloys.

## 2. Materials and Methods

A single-phase brass CuZn36 alloy, characterized by high susceptibility to cold forming, was selected for the tests. The material as delivered was in the form of a drawn bar with a diameter of 8 mm. Before the extrusion, to eliminate the hardening effects, the test material was subjected to recrystallization annealing at a temperature of 550 °C for one hour and then cooled in a furnace to room temperature. The material after such pretreatment is referred to in this paper as the initial material.

The CCE system resembles ECAP by its quadruple multiplication (as ╬). However, the deformation of the workpiece in the cross channel differs from that of the conventional single channel [18,53,54]. The solution is based on the use of a cross matrix channel system, in which the material is compressed in two opposite directions (along the Y-axis in Figure 1), causing the system to reorganize and outflow in two opposite directions that are perpendicular to the original directions (along the X-axis). Depending on the design variant of the device, the workpiece can be deformed again by forcing in the opposite direction into a vertical channel or by turning the entire die to a vertical position and re-extrusion. It is also possible to remove the sample and change the deformation plane by rotating it before placing it back into the die channel.

**Figure 2:**
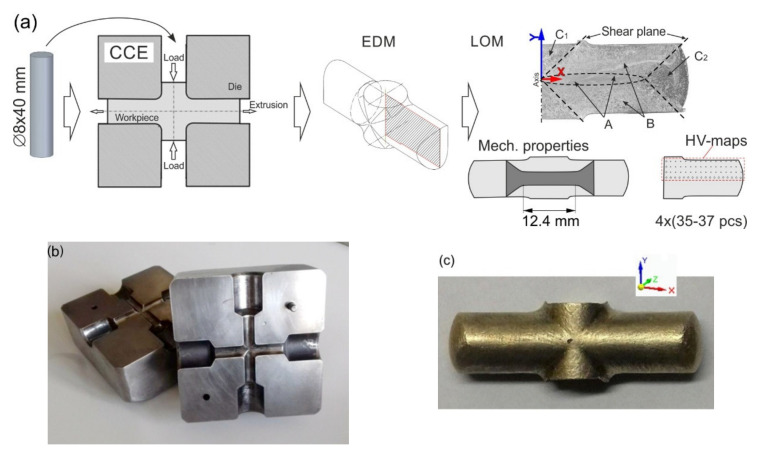
Illustration of CCE process: scheme (**a**), die (**b**), and specimen after processing (**c**).

The tool used for the CCE process (Figure 2a) consists of two equal 1.2080 steel blocks, 160 × 160 × 80 mm in size, that both have a cross-shaped route with a circle shape in cross-section (Figure 2b). Both die halves were closed with steel frames with wedge-shaped pressing surfaces. The diameter of channels is 8 mm and 50 mm in length. The cross channels are connected by an arc having a radius of 2 mm. Two cylindrical punches are used to press the material, and the other two to delay (block) the outflow of the material from the die. The whole is mounted on a device responsible for the uniform movement of both pistons in relation to the die. The extrusion was carried out on a single-action press.

A sample of CuZn36 alloy with dimensions of ∅8 × 40 mm was pressed in a die with a cross channel 2, 4, 8, and 12 times without rotation and two and four times with unidirectional rotation by 90° after each pass. In the first case, it corresponds to the A route, while in the second, it corresponds to B_C_. In variant A, the material is sheared in odd culverts (Figure 1a), while in even-numbered culverts, its cell is straightened by strain reversal. However, in the B_C_ variant, in the first two passes, the cell is sheared successively in two different planes, creating a triclinic cell and then straightens in two successive passes (Figure 1b). The analysis of the presented diagrams also allowed the authors to conclude that the C-route does not occur in the CCE method, as it is the same as the A route. Since the die channel is circular, in the B route, the excess material remaining from the previous pass in the center of the sample was removed mechanically before being placed back into the die (Figure 2c). This allowed the sample to be restored to the original shape of the cylinder. A dry graphite lubricant spray was used to reduce the friction at the metal–tool interface.

The samples were cut parallel to the extrusion direction (X-Y) from the central parts by an electrodischarge machining (EDM) device and then ground on SiC paper with a gradation of 100–4000, polished with 3–0.25 μm diamond suspensions and etched with the “Kalinge’s” reagent (50 mL CH_3_OH, 50 mL HCl, 5 g CuCl). The microstructure was analyzed using a Nikon MA200 metallographic microscope (Irvine, CA, USA), and the grain size was determined using the dedicated computer program NIS-Elements by Nikon.

The mechanical properties of the samples were evaluated using an Instron 8801 testing machine (High Wycombe, UK) with two speeds: 1 mm·s^−1^ for a 0–1 mm and 5 mm·s^−1^ for the remainder of the displacement at room temperature. The classic dumbbell-shaped samples had dimensions of 17 × 3.5 × 1.2 mm and were used for the measurements. The samples were cut so that their measurement section included the material zone on both sides of the X-axis. This corresponds to the zone of the greatest deformation in structures A and partly B, as shown in Figure 2a. The load force-elongation data were recorded by tensile tests carried out on the specimens with a gauge length of 12.5 mm. After each pass, Vickers microhardness measurements were conducted on vertical-longitudinal sample sections using a 200 G load (HMV-G, Shimadzu, Japan). Measurements were made on ¼ of the area of the longitudinal Section X-Y along four paths, starting at a distance of 0.2 mm from the outer surface and ending on the longitudinal X. Each time, 120–150 measurement points were obtained. On their basis, the Origin^®^ (OriginLab Corporation, Northampton, MA USA) program developed contour maps of the microhardness distribution. They provide a visualization of the hardness and homogeneity distribution in the billet by assigning a given level to a specific color.

## 3. Results and Discussion

Figure 2c is a view of a CCE pressed sample. A characteristic arrangement of furrows (>> <<) can be observed on the outer surface, inclined to the X-axis at an angle of approximately 45°. It represents the movement of the material through the shear planes. No cracks were observed on the A-CCE outer surface of the samples, regardless of the number of passages, which occurred in variant 4× in B_C_-CCE.

**Figure 3:**
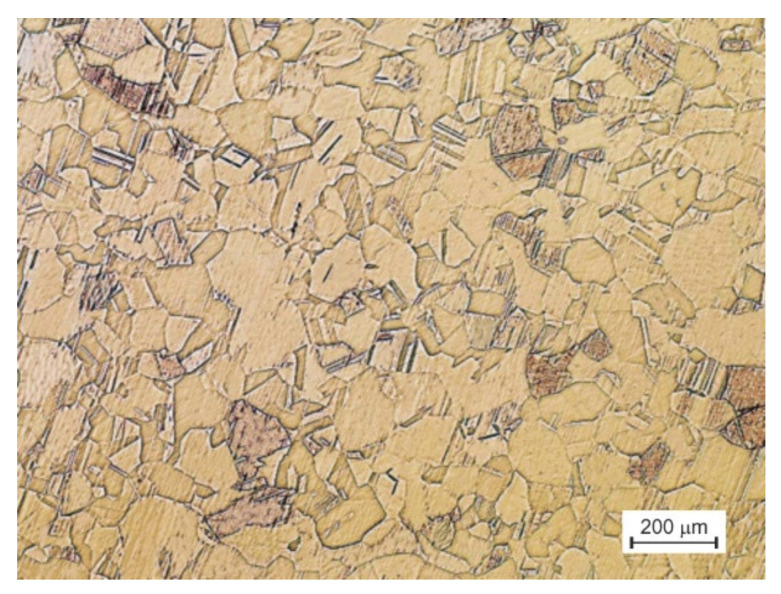
Initial microstructure of CuZn36 (% at.) alloy.

The material structure in its initial state, i.e., after recrystallization annealing, is shown in Figure 3. It is characterized by an equiaxed grain with an average size of 64 µm.

**Figure 4:**
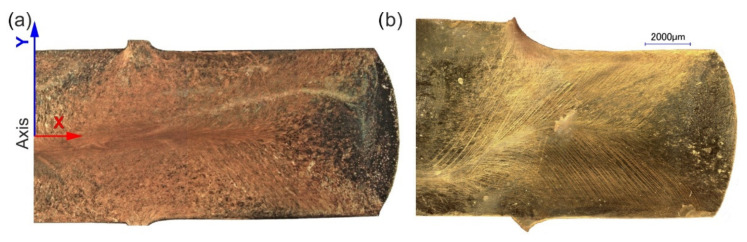
Macroscopy view of CCE specimens (½) after two passes in the A (**a**) and Bc (**b**) routes.

Figure 4 shows the macrostructure images of the samples after being extruded by the CCE method. Visible disturbances in the symmetry of the structure result from incomplete synchronization of the piston movement and the stop. From a technological point of view, this fact did not have a significant impact on the final result of the process, as it is dispersed in the next pass.

**Figure 5:**
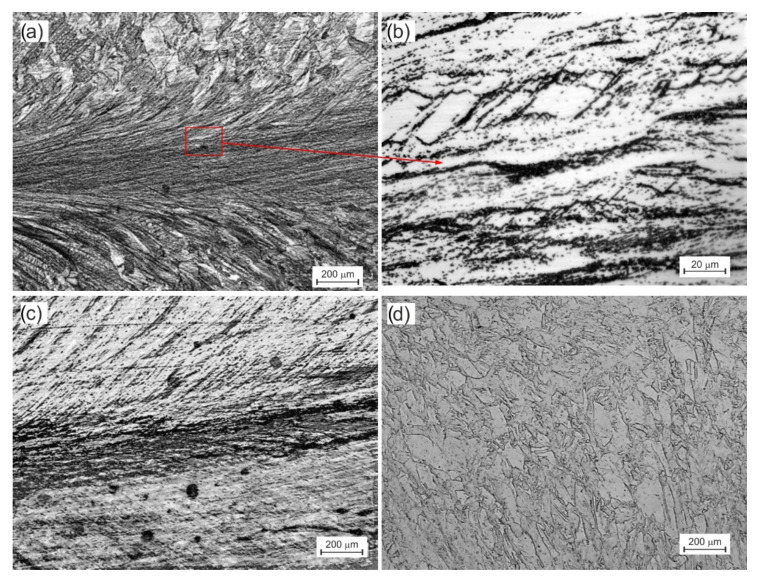
Light microscopy microstructure of A route CCE processing samples after two passes (**a**,**b**) and 12 passes (**c**,**d**). The pictures show the central part of the sample, along *X*-axis, with the structure of A and B zones (**a**–**c**) and C zone (**d**).

Three types of structures were distinguished related to their location (see diagram in Figure 2a). The first (A), with a very strong deformation, creates a characteristic band along the *X*-axis of the extruded sample (Figure 5a). It was shaped due to the interaction of compressive and shear stresses. The grains were strongly elongated with a blurring of the boundaries. For 2× in A-CCE, the width of these bands and, therefore, the grains did not exceed 15 μm (Figure 5b).

A second large B zone associated with shear stresses was created on both sides of the band A zone. The structure of the material was deformed with many slip bands. The grains (60 µm) were slightly distorted toward the outer surfaces, related to passage through the shear plane. As the number of passes increases, the deformation of the material increases. As shown in Figure 5c, this results in a homogeneous structure in each of the zones but also leads to blurring of the grain boundaries. For this reason, determining their size using stereological methods has become unreliable. The strand structure (A) was limited, and the thickness of the individual strands decreased. However, zone B is enlarging and homogenizing at the same time. The third tapered C-zone (Figure 5d) associated with blocked stresses was observed directly under the die piston (C1) or on the newly formed foreheads of the sample (C2). Their grains underwent much less deformation because they were not subject to the two previously mentioned deformation mechanisms.

**Figure 6:**
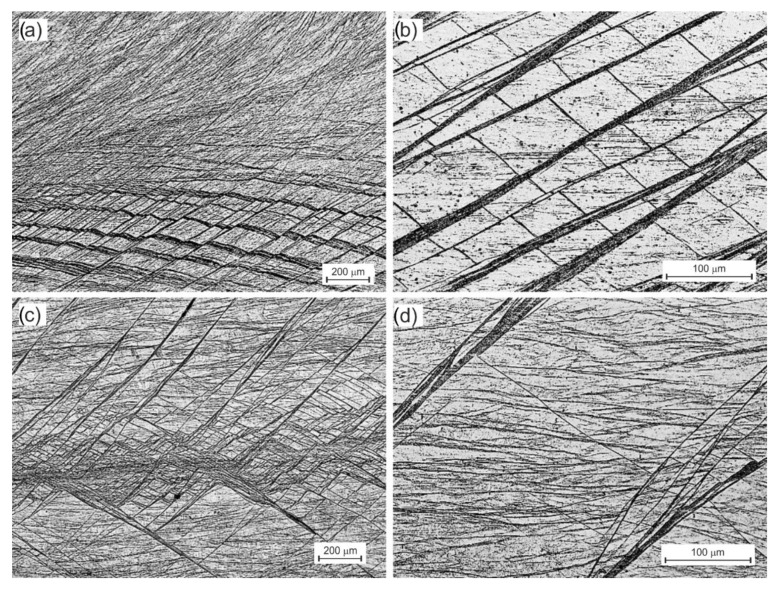
Microstructure of Bc route CCE process: two passes (**a**,**b**) and four passes (**c**,**d**). The structure of A and B zones.

This process was slightly different in B_C_ route. As a result of the rotation of the sample by an angle of 90°, the shear bands formed in the first pass intersected with new second pass bands (Figure 6a). While in zone An, it has not caused any discernible differences, in zone B, it has. The intersecting strands form equilateral cells, 20 × 30 µm (Figure 6b) and larger. As they move away from the X-axis and zone A toward the outer surface, their size increases rapidly, and they begin to resemble those 2× in A-CCE. The subsequent two passes deepen this process (Figure 6c). A further division of the structure with the formation of orthorhombic cells with a side even slightly above 1 µm was observed, or the initiation of the process of dynamic reconstruction structure in the areas defined by shear bands (Figure 6d).

**Figure 7:**
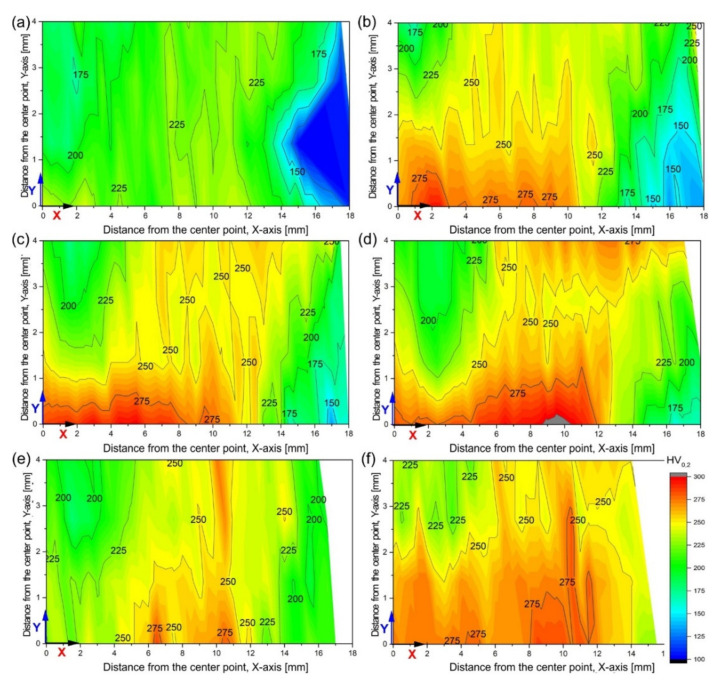
Evolution of microhardness in CCE process: 2× (**a**), 4× (**b**), 8× (**c**), 12× (**d**) in A route and 2× (**e**), 4× (**f**) in B_C_ route. The maps concern one-fourth of the sample area delimited by the X and Y axes, according to scheme in Figure 2a.

The hardness of the tested alloy in the initial state (recrystallized) was 86 HV0.2. The changes in the microhardness of the investigated alloy after deformation are presented as macromaps covering ¼ of the longitudinal section area (X-Y) of the sample (Figure 7). The lower and left edges of the map correspond to the positions of the X and Y axes, respectively, the upper edge of the outer surface, and on the right side, there was a contour corresponding to the newly formed surface of the sample face. In the A route variant of deformation, after only two passes, it increased rapidly to values above 200 HV0.2 with single bands above 225 HV0.2 (Figure 7a). Only in zone C1 was it approximately 175 HV0.2, and C2 was below 125 HV0.2. The strain reversal and the Bauschinger effect can be seen in action. Even though the stresses acting in zone C1 were “blocked”, the material still strengthens. However, the tensile stresses acting on the new sample face in zone C2 lead to a much smaller hardening or even a decrease compared with the previous processing cycle. As the number of passes increases, it can be seen that the process of shaping the paraxial highly deformed zone with microhardness even above 275 HV0.2 and gradual migration of the strengthening toward the outer surface of the sample in zone B (Figure 7b–d).

In the case of deformation in the B_C_ route, the material’s behavior was similar to that in the previous case, but the obtained microhardness values were greater for the same number of passes. It can be said that the material strengthening after two passes in the B_C_ variant corresponds to 4× in the A-route, and 4× in B_C_ was close to the 12× A-route. Additionally, the strengthening in zone C is generally higher, and they are quickly reduced by homogenization with the rest of the material.

**Figure 8:**
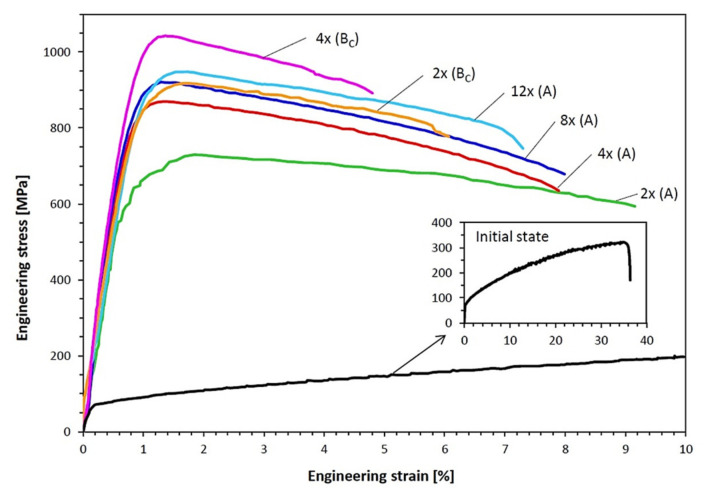
Stress-strain curves for initial and CCE-processed Cu-Zn alloy in the A and B_C_ routes.

Table 1 lists the tensile properties of the CuZn36 alloy that underwent 2–12 passes in the A route and 2–4 passes in the B_C_ route of the CCE process. Additionally, the results of the initial state are also shown. Figure 8 shows the representative engineering stress–strain curves for each condition. The values of the ultimate strength (UTS), yield strength (YS), total elongation (A), and YS/UTS ratio were obtained from the engineering stress–strain curves and the tested samples.

The yield point and tensile strength showed a substantial increase from 76 and 307 MPa to 650 and 730 MPa, respectively, after two passes in A-CCE. These results, combined with a drastic decrease in elongation to ~9.8%, indicated a strong deformation strengthening of the material. The increase in deformation leads to a further increase in these parameters, even up to 920 and 948 MPa. This is equivalent to an almost threefold increase from the initial state. However, in the B_C_-CCE variant, the material shows an even faster and greater increase in strength at the expense of its elongation. 2× in B_C_-CCE is higher than 8× in A-CCE and is close to 12× in A-CCE.

Regardless of the variant of the deformation route, the material reached very high strength parameters after just two passes. High YS/UTS ratio values indicated a low yield point of the material and the need for even partial recrystallization of the alloy.

Damavandi et al. [28] analyzed ECAP patterns of distortion and found that deformation according to the B_C_ route was a process of excess stress. The slip in the first and second passes was limited by the slip in the third and fourth passes, respectively. Therefore, the even passes showed lower strength than the odd passes. However, in route A, different shear planes provide additional deformation and increase in strength more than in the case of deformation according to the B_C_ variant. However, the intersection of planes and shear directions in B_C_ results in more complex systems and may result in higher strength and ductility of the processed material, as demonstrated in this paper for the CCE method.

According to the model proposed by Sakai [25], shear bands and micro bands develop and cross each other to form equiaxed structures at intersections and along the bands. Further processing increases their density, leading to the formation of a fine-grained equiaxed structure. The minimum grain size in SPD processes was determined by developing a dynamic equilibrium between the generation of dislocations and their recovery or the process of refining the grain and the process of its growth [7].

Changing the sample orientation between successive passes, therefore, leads to different combinations of grain shape and size, as shown in Figure 5 and Figure 6. The intersecting slip planes in variant B_C_ divide the grain much more efficiently than in variant A. This resulted in a much faster fragmentation of the structure. Processing with a variable deformation route thus improves the homogeneity [21]. Often nonhomogeneous primary processes had a significant effect on grain size. It should also be noted that the processing of the material with the CCE method leads to a rapid increase in the number of structural defects in the material. As shown in previous studies [54], in the case of copper samples extruded 4× by the CCE method, the stored deformation energy of the material caused its recrystallization temperature to drop to 158.5 °C. Therefore, the defects observed in the case of 4× in B_C_ should be related to the beginning of transformations initiated by dynamic recrystallization of the deformed material [56]. It also creates opportunities for further improvement of the material properties due to heat treatment.

Observations of the material strengthening visualized with the help of contour maps of microhardness distributions significantly facilitate the analysis of the deformation process and change its route to the process of homogenization of the material structure. The obtained results show that processing with SPD methods leads to a high level of hardness in the first passes [57], which increases with a decrease in grain size. Repeating this process with a changing deformation route does not result in a significant increase in its hardness but primarily leads to an improvement in the homogeneity of its structure and properties.

The refinement of the grain as a result of processing with the CCE method causes the alloy, plastic in its initial state, to achieve a very high strength after processing at the level of good-quality structural steel. This was accompanied by a substantial increase in the YS/UTS ratio and a decrease in the plasticity reserve of the material. From the constructor’s point of view, this is a disadvantage, but it may turn out to be an advantage in specific applications.

Considering the abovementioned, it seemed appropriate to utilize advanced techniques for characterizing the material’s structure, such as SEM-EBSD, TEM electron microscopy, or XRD texture analysis. The author intends to supplement this area of knowledge in the near future.

The change of route deformation can be realized not only by the rotation of the sample but also by changing the geometrical parameters. Li et al. [58] modified the classic forward extrusion (CFE), consisting of the use of two semicircular pistons instead of one (AFE-alternate forward extrusion). The alternating stepwise movement of the punches divides the volume of the squeezed batch into active and passive parts. As a result, the deformation zone resembles the letter “Y” with the following deformation sequences alternating on the left and immediately on the right branch zone. At the boundary of these zones, the material undergoes an additional shear deformation, which leads to a prerefining of the microstructure of the blank, which is further refined as it flows through the die opening. Compared with the conventional extrusion, this results in a smaller and more uniform grain.

Quite recently, Zhuo et al. [59] presented the results of studies on the deformation of the AA1050 aluminum rod using the differential velocity sideways extrusion (DVSE) method. In its assumptions, this is a process very similar to the CCE, except that the tool has only one exit channel. Its purpose is to produce a rod with improved structure and arcuate shape, which is helped by the possibility of differentiating the advancing speed of both counter-rotating pistons. Due to the greater deformation (ε = 2–3) than is the case for conventional one-pass equal channel angular extrusion, the material has a significantly refined grain, where its size decreased from 357 μm to ~3 μm in one operation extrusion. Moreover, in the material zone along the sample outflow axis, they observed the presence of a coarse crystalline band structure containing subgrains with low angular boundaries, which gradually transformed into a fine equiaxed subgrain structure with a mixture of low and high angle boundaries. The same authors in [60], while examining the industrial process of manufacturing a shaped profile made of pure aluminum, showed the superiority of the side extrusion process compared to the forward CE. As they argue, greater grain refinement is possible due to the greater effective deformation associated with a radical change in the direction of the material flow. The occurrence of shear stresses resulted in an improvement in the mechanical properties of the processed material in a single pass (increase in YS by 79% and UTS by 80%) in relation to the billet material. At the same time, these values were 10–20% higher than for forward extrusion carried out under the same conditions but without changing the route deformation.

## 4. Conclusions

This study presented the results of processing the CuZn36 (at.%) alloy with a less popular method of extrusion in the CCE cross channel (Supplementary Materials), using an innovative approach changing the deformation route in the A (here equivalent to C) and B_C_ routes.

The CCE method was related to the ECAP method. However, due to the shape of the intersecting channels, deformation is a more complex process. The CCE method eliminates the problems found in ECAP, such as channel bottom friction or “corner gaps”, but requires greater demands on the control of the process. The basic deformation method was also shear, but a significant share of triaxial compression leads to the formation of a paraxial zone of very strong deformation. It is a potential source of structural changes. Unfortunately, its presence increases the heterogeneity of the material.

The increase in deformation resulting from the multiplication of the number of passes of the billet through the matrix system, regardless of the adopted pattern of the deformation route, leads to a substantial strengthening of the alloy. The effect of this was also an increase in microhardness, its propagation throughout the sample volume, and an increase in the homogeneity of the structure. The observed decrease in grain size was also responsible for the increase in strength.

In the A route, processing of the material leads to a substantial increase in the strength properties of the material, with a clear division into the types of its structure. In the B_C_ route, as a result of rotating the sample at an angle of 90°, the deformation planes intersect, generating the shear bands and microbands that efficiently divide the grain into parallelogram grains. The amount of energy accumulated in this way was so significant that the beginning of dynamic recrystallization was observed. The strain efficiency in the B_C_ route was much greater than that in A. It can be said that processing 2× in B_C_ by CCE corresponds to 8× in A, and 4× in B_C_ gives higher values than 12× in the A route.

## Figures and Tables

**Table 1:** Tensile properties of CuZn36 (% at.) alloy in the initial state and after CCE.

Route	Number of Passes	YS MPa	UTS MPa	A %	YS/UTS
Initial–annealed		76	307	78	0.25
A	2	650	730	9.8	0.89
	4	830	870	8.0	0.95
	8	882	921	8.0	0.96
	12	920	948	7.5	0.97
B_C_	2	865	917	6.0	0.94
	4	1018	1042	4.8	0.98

## Supplementary Materials

The following supporting information can be downloaded at: https://www.mdpi.com/article/10.3390/ma15031124/s1, Video S1: CCE method by Łyszkowski.
